# Haematology and biochemistry reference intervals of Galapagos short-eared owls (*Asio flammeus galapagoensis*) from Floreana Island

**DOI:** 10.1093/conphys/coag013

**Published:** 2026-03-02

**Authors:** Emma Vaasjo, Ainoa Nieto-Claudin, Paula A Castaño, Gislayne Mendoza-Alcívar, Birgit Fessl, Vivian Huacuja García, Kathleen Apakupakul, Sharon L Deem

**Affiliations:** Wilder Institute/Calgary Zoo, Department of Animal Care, Health, and Welfare, 300 Zoo Road NE, Calgary, AB T2E 7V6, Canada; Saint Louis Zoo Institute for Conservation Medicine, 1 Government Drive, Saint Louis, MO 63110, USA; Saint Louis Zoo Institute for Conservation Medicine, 1 Government Drive, Saint Louis, MO 63110, USA; Charles Darwin Research Station, Charles Darwin Foundation, Charles Darwin Avenue, Santa Cruz, Galapagos Islands 200350, Ecuador; Island Conservation, Charles Darwin Avenue, Santa Cruz, Galapagos Islands, Ecuador; Charles Darwin Research Station, Charles Darwin Foundation, Charles Darwin Avenue, Santa Cruz, Galapagos Islands 200350, Ecuador; Charles Darwin Research Station, Charles Darwin Foundation, Charles Darwin Avenue, Santa Cruz, Galapagos Islands 200350, Ecuador; Island Conservation, Charles Darwin Avenue, Santa Cruz, Galapagos Islands, Ecuador; Saint Louis Zoo Institute for Conservation Medicine, 1 Government Drive, Saint Louis, MO 63110, USA; Saint Louis Zoo Institute for Conservation Medicine, 1 Government Drive, Saint Louis, MO 63110, USA; Charles Darwin Research Station, Charles Darwin Foundation, Charles Darwin Avenue, Santa Cruz, Galapagos Islands 200350, Ecuador

**Keywords:** Haematology; *Asio flammeus galapagoensis*, biochemistry, conservation, Galapagos, Galapagos short-eared owl, reference interval, sex difference

## Abstract

To limit the impact of invasive predator species on the ecosystem of Floreana Island, Galapagos, a large-scale rodent eradication program was implemented. Due to the significant risk of secondary exposure to rodenticide, a mitigation tactic was used to protect the only native raptor found on Floreana, the Galapagos short-eared owl (*Asio flammeus galapagoensis*). A large proportion of the owl population was brought into human care in July 2023 with the goal to release back to the wild following the completion of the eradication program and reduction of the risk of exposure to rodenticides. During this time under human care, health examinations including blood collection for assessment of haematology and biochemistry parameters were completed. Descriptions of leukocyte morphology and population-based reference intervals (RI) are reported here utilizing results from 62 clinically healthy adults. Sex was determined using polymerase chain reaction, allowing for comparisons between males (*n* = 29) and females (*n* = 33). Statistically significant differences were found for several parameters: packed cell volume, relative and absolute monocyte counts, total protein, calcium and phosphorus levels were higher in females, while uric acid and glucose levels were significantly higher in males. These baseline RI are an important tool for assessment of this unique population while under human care, and will allow for comparisons and continued health monitoring after release back to the island of Floreana.

## Introduction

Floreana is the smallest inhabited island in the Galapagos archipelago and is currently home to a single endemic raptor subspecies, the Galapagos short-eared owl (*Asio flammeus galapagoensis*) (DeGroot, 1983; Dvorak *et al*., 2017; Schulwitz *et al*., 2018). Formal population estimates of Galapagos short-eared owls throughout the archipelago have not been done since the 1980s (DeGroot, 1983), although more recent surveys on Floreana show evidence of declining short-eared owl populations, in addition to the extirpation of sympatric land birds (Dvorak *et al*., 2017). The Island’s fragile ecosystem has been significantly altered by the effects of human habitation and the introduction of invasive predator species such as rodents and feral cats, which have been directly related to population declines and extirpation of native species (Phillips *et al*., 2011; Bellard *et al*., 2016; Dvorak *et al*., 2017). To address this, a large scale and complex invasive vertebrate eradication program was initiated, resulting in the eradication of goats and donkeys from Floreana Island after a focused effort from 2006 to 2009 (Carrion *et al*., 2011). Further efforts to remove invasive species using the anticoagulant rodenticide brodifacoum are currently underway, with the goal of reducing populations of black rats (*Rattus rattus*), house mice (*Mus musculus*) and feral cats. Brodifacoum is a second-generation anticoagulant known for being highly effective for rodent eradication on islands but can present risk to non-target species, through primary and secondary pathways of exposure (Hoare and Hare, 2006; Castaño *et al*., 2022). The population of Galapagos short-eared owls on Floreana Island was considered a non-target species at high risk of rodenticide exposure mortality (Rueda *et al*., 2016); therefore, a mitigation plan to protect short-eared owls on the island was implemented.

Although short-eared owls are found throughout the Galapagos Islands, owls on Floreana display unique behaviours when compared to other island populations. They are more crepuscular due to a lack of other raptors on the island, mainly the diurnal Galapagos hawk (*Buteo galapagoensis*) (DeGroot, 1983), and are less responsive to play back calls recorded from populations on neighbouring islands (Schulwitz *et al*., 2018). These unique traits, as well as genetic studies, have resulted in the species being identified as an Evolutionary Significant Unit (personal communication from Dr. Hywel Glyn Young, Durrell Wildlife Conservation Trust), therefore requiring mitigation to be implemented to preserve their unique genetics and behaviours. The mitigation tactic selected was to house a proportion of the Floreana short-eared owl population under human care on a neighbouring island, Santa Cruz, for a period long enough to avoid significant mortality from secondary poisoning.

This method allowed for an opportunity to perform comprehensive health assessments on a significant proportion of this unique population of short-eared owls. The only published reference range for haematology and biochemistry of short-eared owls was determined after sampling a population of the nominate subspecies (*Asio flammeus flammeus*) held under human care in France and Canada (Ammersbach *et al*., 2015a, 2015b). Genetic assessments estimate that the Galapagos short-eared owl diverged from this more broadly distributed subspecies found in North America, Europe, northern Africa, and Asia around one million years ago (Schulwitz *et al*., 2018). Although research has been published on population numbers and distribution, genetics, behaviour and life history of the Galapagos subspecies (Dvorak *et al*., 2017; Schulwitz *et al*., 2018; Ludica *et al*., 2021), little is known about their health status. Accurate, population-based RI are critical for assessing health status and are an integral component to conservation programs (Deem *et al*., 2001). The objective of this study was to establish haematology and biochemistry RI for this population, including potential differences between males and females, to support future interpretation and monitoring of diagnostic sampling.

## Materials and Methods

### Study population

Owls were captured on Floreana Island between July and August of 2023. A total of 65 owls were captured utilizing either rodent-baited bal-chatri traps (Bloom *et al*., 2007) or an extendable capture tube with a rope tied into a slip knot as described in a previous study of raptors in the Galapagos (Deem *et al*., 2012). All owls were examined upon capture to ensure they were in good health and were treated for external and internal parasites prior to transport to follow Galapagos Biosecurity Agency protocols for animal transportation between islands. This study was conducted under Scientific Research Permits issued by the Galapagos National Park Directorate (PC-56-23, PC-45-24 and PC-29-24).

Owls were then housed in a dedicated facility located in an area chosen to minimize human contact within the Galapagos National Park on Santa Cruz Island. Housing consisted of ten aviaries within a single building ranging in size from 8 × 4 × 2.8 m to 15 × 4 × 2.8 m. Owls were kept in mixed-sex groups ranging from 5 to 10 individuals per aviary. The aviaries were constructed to allow for natural light, airflow and visual access to the surrounding environment, but were fully enclosed with mesh to prevent access of invasive species (rodents, cats, dogs, etc.). The whole prey, pre-killed diet consisted primarily of one-day-old chicks (1–2 chicks per owl per day, sourced from Avicola San Isidro S.A., Isidro Ayora, Ecuador), occasionally supplemented with rats (captive bred on site). Fresh water was available *ad libitum.* Owls were also offered a commercial vitamin supplement (Vitahawk Maintenance, Vitahawk, Oakley CA, USA) formulated for raptors once a week. Throughout the period under human care, specific measures were implemented to safeguard physiological health and maintain species-typical behaviours. These included structured husbandry protocols that limited human interaction, environmental enrichment (varied perching, dust and water baths, misting, substrate rotation, etc.), and regular flight conditioning.

### Sample collection

Following pre-established protocols for care of the owls, all animals underwent a physical examination at the time of sample collection in January 2024 (6–7 months following the transition to human care). Examinations included collecting an updated body weight and estimating a body condition score (BCS), which was assigned on a scale of 1 to 5 by palpating the musculature over the keel and assessing for fat deposits around the pelvic limbs, axilla and neck (a score of 1 indicates a thin bird with underdeveloped musculature and no fat deposits, 3 is a bird in ideal condition and 5 indicates a significantly overconditioned bird with considerable subcutaneous fat deposition). We also performed recheck assessments for the presence of ectoparasites and the nematode *Serratospiculum tendo*, which can be identified visually in the subcutaneous space around the external ear, at this time. We collected a minimum of 0.3 ml of blood from the medial metatarsal vein of each owl using a 1-ml heparinized syringe and a 23–25 G, 1-inch needle. Two blood films were made immediately with whole blood, and once dried, these were fixed with methanol (Fixative 1, Jorvet™ Diff Quick Stain Kit, Jorgensen Laboratories, Loveland CO, USA) prior to transport to the laboratory for haematology. One drop of blood was saved on an FTA Mini card (Whatman) for sexing, and the remainder was placed in a lithium heparin microhematocrit tube and stored at 4°C for a maximum of 4 hours before further hematologic and biochemical analysis. All blood samples were collected within the first 15 minutes of handling time.

### Haematology and biochemistry

Packed cell volume (PCV) was determined after high-speed centrifugation of microhematocrit tubes filled with heparinized whole blood using a ZIPocrit Reader Card (LW Scientific, Lawrenceville GA, USA). Total solids (TS) were determined using plasma on a refractometer (Jorvet™ J-351 Clinical Refractometer, Jorgensen Laboratories).

Modified Wright Giemsa stain (Jorvet™ Diff Quick Stain Kit, Jorgensen Laboratories) was used to stain blood films initially made during blood collection by following the manufacturer’s instructions. Slides were reviewed to describe white blood cell (WBC) morphology and to perform a differential WBC assessment. An experienced veterinarian (ANC) counted 100 WBCs per animal, and results were used to calculate relative percentage and absolute counts of heterophils, lymphocytes, eosinophils, monocytes and basophils, as well as the heterophil to lymphocyte ratio (H:L).

Total WBC count was determined using the phloxine B method. Briefly, 25 μl of whole blood from the lithium heparin tube was placed into a pre-filled tube of 0.1% phloxine (775 μl, Leukopet, Jorgensen Laboratories). This solution was used to charge the haemocytometer, and all stained cells (heterophils and eosinophils) were counted in both chambers. The total leukocyte count was then calculated using the following formula (Campbell and Grant, 2022b):


\begin{align*} &\mathrm{Total}\ \mathrm{WBC}/\mathrm{\mu} \mathrm{L}\\&=\frac{\mathrm{total}\ \mathrm{heterophils}+\mathrm{eosinophils}\ \left(\mathrm{both}\ \mathrm{chambers}\right)\ \mathrm{x}\ 1.1\ \mathrm{x}\ 16\ \mathrm{x}\ 100}{\%\mathrm{heterophils}+\%\mathrm{eosinophils}\ \left(\mathrm{from}\ \mathrm{differential}\ \mathrm{count}\right)} \end{align*}

Biochemistry was assessed using VetScan® Abaxis Avian/Reptilian rotors (Zoetis, Parsippany NJ, USA) on whole blood from lithium heparin tubes within 4 hours of collection. The biochemical panel included sodium (Na), potassium (K), calcium (Ca), phosphorus (P), uric acid (UA), aspartate aminotransferase (AST), creatinine kinase (CK), glucose (Glu), total protein (TP), albumin (Alb), globulin (Glob) and bile acids (BA).

### Molecular sexing

Blood samples preserved on FTA cards underwent DNA extraction using the DNeasy Blood & Tissue Extraction Kit (Qiagen, Germantown MD, USA) utilizing the protocol described in the QIAamp® DNA Micro Handbook (Qiagen). To genetically determine the sex of the owls, markers on the Z and W chromosomes were amplified via polymerase-chain reaction (PCR) (Itoh *et al*., 2001; Griffith *et al*., 2024). Each 24.5 μl of PCR reaction mixture consisted of 12.5 μl Amplitaq Gold™ 360 Master Mix (Thermo Fisher Scientific, Waltham MA, USA), 8 μl nuclease-free water, 1.0 μl of each primer (10 μM) and 2 μl aliquot of DNA template. For the second amplification in the nested PCR assay, we used the same protocol above, with a 2 μl aliquot from the first reaction. Each sample was subjected to two consecutive PCRs to amplify a 470 bp region of the W chromosome (only females) and a 250 bp region of the Z chromosome (males and females) as follows: 94°C 5 min; 34x (95°C 80 s, 52°C 90 s, 72°C 60 s); 72°C 5 min (Griffith *et al*., 2024). All PCR products were resolved on 1% agarose gels for electrophoresis (120 V, 70 min) and visualized using Sybr Safe DNA gel stain (Thermo Fisher Scientific) under ultraviolet light. Samples amplifying for both W and Z (control) chromosomes were considered females whereas the amplification of only the Z chromosome was considered male.

### Statistical analysis

The ASCVP has laid out standardized guidelines for the determination of RI in veterinary species (Flatland *et al*., 2013). Non-parametric or robust methods are recommended when sample sizes fall between 40 and 120. Utilizing these guidelines and Reference Value Advisor (RefVal v2.1), descriptive statistics (mean, median, SD, min and max), 95% RI and 90% confidence intervals (CI) were determined for each analyte.

The Anderson–Darling test and histogram evaluation were used to assess symmetry and data distribution. As this study is based on a smaller sample set, a threshold *P*-value of 0.3, instead of the more typical .05, was used to more accurately characterize the results (Flatland *et al*., 2013). Therefore, if the test’s *P*-value was <0.3, the analyte was determined to have a non-Gaussian distribution. Outlier values were also identified in RefVal using Dixon and Tukey’s range tests. Each suspected outlier was closely reviewed and only those attributed to poor sample quality or analytic error were manually removed.

All parameters were assessed for normality using the Kolmogorov–Smirnov test. We compared body weight, biochemistry and haematology parameters between sexes using the Mann–Whitney U Test (Wilcoxon Rank Sum Test) as the criteria for Gaussianity was not met. All analyses were performed using IBM® SPSS Statistics 25 and used *P* < .05 for all tests other than Anderson-Darling as noted above.

## Results

Of the 65 owls sampled for this study, a total of 62 were considered clinically healthy based on examination. The remaining three animals, although generally in good health, were receiving antiparasitic treatment for *S. tendo* at the time of sampling and were therefore excluded from RI calculations. Results from molecular sexing confirmed the study population consisted of 33 females and 29 males. The mean (± SD) weight for males was 384.0 ± 21.4 g and 440.6 ± 30.3 g for females; females were significantly heavier than males (*P* < .001). Body condition ranged from 3.0 (ideal condition) to 5.0 (significantly overconditioned) with a median of 3.50 (IQR 3.25–3.94). Lesions associated with class 1 pododermatitis (Remple, 1993) were present in a subset of owls on examination (*n* = 21); these animals were included in RI calculations as lesions were determined to be mild and no significant differences were observed in blood values when animals with and without pododermatitis-like lesions were compared.

We present the RI for haematology and biochemistry of Galapagos short-eared owls in Table 1. Errors were occasionally reported for individual analytes on the VetScan biochemistry results, most commonly for total protein, albumin and globulins. Bile acid results were below the limit of detection (<35 μmol/L) for all samples, and therefore not included in further analyses. Values for calcium and potassium were also occasionally reported as beyond the detection limits of the analyser, two samples returned Ca values as > 16 mg/dl, and three samples returned *K* values as <1.5 mmol/L. In these cases, conservative alterations of the data were made to calculate RI, and results were rounded to the nearest numeric value (16.1 mg/dl for Ca, 1.4 mmol/L for K). Similar methods have been used previously in RI determinations using this analyser (Nieto-Claudin *et al*., 2021).

**Table 1:** RI and CI of haematology and plasma biochemistry parameters for Galapagos short-eared owls (*Asio flammeus galapagoensis*)

**Analyte**	**SI units**	***N***	**Mean**	**SD**	**Median**	**Min**	**Max**	***P***	**Distribution**	**Method**	**LRL of RI**	**URL of RI**	**CI 90% of LRL**	**CI 90% of URL**	**Outliers**
PCV	L/L	62	46	2.9	46	40	54	0.048	NG	NP	40.0	52.3	40.0–42.0	50.0–54.0	0
TS	g/L	61	42.6	4.2	42.0	32.0	52.0	<0.001	NG	NP	34.2	50.9	32.0–38.0	50.0–52.0	0
WBC	10^9^/L	60	10.3	4.5	9.7	3.8	24.3	0.036	NG	NP	3.9	22.5	3.8–4.6	17.6–24.3	2
Heterophil	%	60	25.2	6.0	25.0	11	37	0.042	NG	NP	11.5	36.0	11.0–14.1	33.0–37.0	2
Heterophil	10^9^/L	60	2.5	1.3	2.3	0.97	7.29	<0.001	NG	NP	1.1	6.5	1.0–1.3	4.8–7.3	2
Lymphocyte	%	60	48.2	8.3	48.5	31	69	0.663	G	NP	32.1	69.0	31.0–34.6	60.0–69.0	2
Lymphocyte	10^9^/L	60	4.9	2.1	4.6	1.4	9.6	0.055	NG	NP	1.8	9.5	1.4–2.1	8.8–9.6	2
Monocyte	%	60	12.1	4.8	11.0	4	25	0.010	NG	NP	5.1	24.5	4.0–6.5	20.5–25.0	2
Monocyte	10^9^/L	60	1.3	1.0	1.0	0.24	5.11	<0.001	NG	NP	0.3	4.6	0.2–0.4	3.3–5.1	2
Eosinophil	%	60	12.6	5.8	11.0	3	33	<0.001	NG	NP	3.5	30.9	3.0–6.1	24.0–33.0	2
Eosinophil	10^9^/L	60	1.3	0.9	1.2	0.17	4.13	<0.001	NG	NP	0.3	3.9	0.2–0.4	3.2–4.1	2
Basophil	%	60	2.0	1.9	1.5	0	7	<0.001	NG	NP	0.0	7.0	0.0–0.0	5.0–7.0	2
Basophil	10^9^/L	60	0.2	0.2	0.1	0	0.68	<0.001	NG	NP	0.0	0.7	0.0–0.0	0.5–0.7	2
H:L	%	60	0.6	0.2	0.5	0.17	1	<0.001	NG	NP	0.2	1.0	0.2–0.2	0.9–1.0	2
Na	mmol/L	61	145	5.3	143	134	157	<0.001	NG	NP	135.1	155.9	134.0–139.0	153.0–157.0	0
K	mmol/L	60	2.2	0.4	2.2	1.4	3.1	0.076	NG	NP	1.4	3.0	1.4–1.5	2.8–3.1	0
Ca	mmol/L	62	2.6	0.4	2.5	2.1	4.0	<0.001	NG	NP	2.1	4.0	2.1–2.2	3.4–4.0	0
P	mmol/L	60	1.8	0.7	1.7	0.6	3.6	0.001	NG	NP	0.7	3.6	0.6–1.0	3.3–3.6	2
UA	μmol/L	62	532.3	230.8	490.7	202.2	1284.8	0.001	NG	NP	212.5	1230.0	202.2–267.7	945.5–1284.8	0
AST	U/L	60	292.7	134.8	239.0	150	773	<0.001	NG	NP	159.5	693.2	150.0–172.7	562.2–773.0	1
CK	U/L	59	248.5	175.1	175.0	77	875	<0.001	NG	NP	80.0	819.0	77.0–88.0	574.5–875.0	1
Glu	mmol/L	62	19.4	2.9	19.3	11.9	26.4	0.414	G	NP	12.1	26.0	11.9–14.7	24.2–26.4	0
TP	g/L	52	35.2	3.2	35.0	27.0	41.0	0.447	G	NP	27.7	41.0	27.0–30.0	40.4–41.0	0
Alb	g/L	52	31.3	3.6	31.5	25.0	40.0	0.202	NG	NP	25.0	39.4	25.0–26.0	37.0–40.0	0
Glob	g/L	52	3.8	3.0	3.0	0.0	13.0	<0.001	NG	NP	0.0	12.7	0.0–0.3	9.7–13.0	0

NP, nonparametric. According to ASVCP guidelines, analytes should be determined as having a Non-Gaussian (NG) distribution if *P* < 0.3. LRL: lower reference limit; URL: upper reference limit; PCV: packed cell volume; TS: total solids, WBC: white blood cell count; H:L: heterophil:lymphocyte ratio; Na: sodium; K: potassium; Ca: calcium; P: phosphorus; UA: uric acid; AST: aspartate aminotransferase; CK: creatinine kinase; GLU: glucose; TP: total protein; Alb: albumin; Glob: globulins.

In Table 2, we present values for male and female owls, highlighting a number of statistically significant differences. Relative and absolute monocyte counts, packed cell volume, total protein and phosphorus were significantly higher in females, whereas uric acid and glucose were significantly higher in males.

**Table 2:** Haematology and plasma biochemistry values for male and female Galapagos short-eared owls (*Asio flammeus galapagoensis*)

		**Female**		**Male**		
**Analyte**	**SI units**	***n***	**Mean ± SD**	***n***	**Mean ± SD**	***P***
PCV	L/L	33	47.0 ± 3.1	29	45.0 ± 2.2	0.009^*^
TS	g/L	33	43.2 ± 4.2	29	42.0 ± 4.3	0.309
WBC	10^9^/L	33	11.2 ± 5.1	29	9.7 ± 3.8	0.287
Heterophil	%	33	25.7 ± 5.8	29	24.4 ± 6.4	0.581
Heterophil	10^9^/L	33	2.8 ± 1.7	29	2.3 ± 1.1	0.143
Lymphocyte	%	33	46.9 ± 6.6	29	48.5 ± 10.6	0.397
Lymphocyte	10^9^/L	33	5.2 ± 2.3	29	4.6 ± 1.9	0.295
Monocyte	%	33	13.4 ± 4.8	29	10.3 ± 4.2	0.006^*^
Monocyte	10^9^/L	33	1.6 ± 1.1	29	1.0 ± 0.7	0.027^*^
Eosinophil	%	33	12.3 ± 4.6	29	14.5 ± 9.1	0.697
Eosinophil	10^9^/L	33	1.4 ± 0.9	29	1.2 ± 0.8	0.471
Basophil	%	33	1.8 ± 1.6	29	2.3 ± 2.4	0.559
Basophil	10^9^/L	33	0.2 ± 0.2	29	0.2 ± 0.2	0.839
Na	mmol/L	33	145.6 ± 4.8	29	144.3 ± 5.8	0.114
K	mmol/L	32	2.2 ± 0.5	29	2.2 ± 0.4	0.669
Ca	mmol/L	33	2.7 ± 0.5	29	2.4 ± 0.2	0.022^*^
P	mmol/L	33	2.2 ± 1.0	29	1.6 ± 0.5	0.028^*^
UA	μmol/L	33	439.1 ± 136.2	29	638.8 ± 270.0	0.002^*^
AST	U/L	32	312.6 ± 144.6	29	290.9 ± 162.2	0.402
CK	U/L	30	271.0 ± 220.0	29	225.2 ± 110.6	0.570
Glu	mmol/L	33	18.4 ± 2.4	29	20.6 ± 2.9	0.001^*^
TP	g/L	30	35.9 ± 3.4	22	34.1 ± 2.7	0.048^*^
Alb	g/L	30	31.4 ± 3.9	22	31.1 ± 3.2	0.970
Glob	g/L	30	4.4 ± 3.3	22	3.0 ± 2.5	0.164

The Mann–Whitney U test was used to assess for significant differences between groups. PCV: packed cell volume; TS: total solids, WBC: white blood cell count; Na: sodium; K: potassium; Ca: calcium; P: phosphorus; UA: uric acid; AST: aspartate aminotransferase; CK: creatinine kinase; GLU: glucose; TP: total protein; Alb: albumin; Glob: globulins.

^*^Values differ between males and females, significant (*P* < .05)

**Figure 1:**
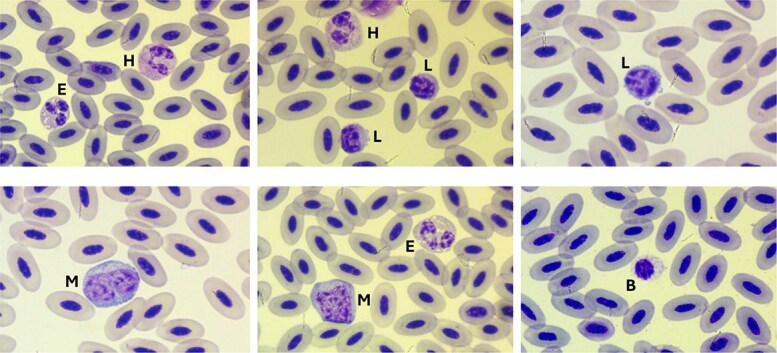
Modified Wright-Giemsa-stained peripheral blood from Galapagos short-eared owls (*Asio flammeus galapagoensis*). H, heterophil; E, eosinophil; L, lymphocyte; M, monocyte; B, basophil.

Morphological characteristics of the WBCs are shown in Fig. 1. Drying artefacts derived from extreme weather conditions and slow dry time were present in some slides. The predominant leukocyte were lymphocytes, characterized by round nuclei with heavily clumped chromatin and scant, often irregular, weakly basophilic cytoplasm. Monocytes were the largest leukocyte identified, with abundant blue–grey cytoplasm. Monocytes had a more irregularly shaped nucleus compared to lymphocytes, though distinct chromatin clumping was still present. Heterophils were mostly round, though occasionally slightly distorted, with a bilobed or trilobed nucleus with clumped chromatin, and clear cytoplasm. Eosinophils appeared very similar, though they could be differentiated from heterophils due to differences in granule appearance. Both cell types contained numerous eosinophilic granules, granules were more darkly stained, pleomorphic (rice-shaped to round), and occasionally refractile in heterophils, and more lightly stained and round in eosinophils. Eosinophils also had a more blue-tinged cytoplasm and often contained numerous clear vacuoles, which also eased differentiation. Basophils were degranulated in all smears with only sparse granules and even more numerous clear vacuoles. We did not detect cell toxicity in any of the individual owls.

## Discussion

In this study, we present baseline haematology and biochemistry RI for Galapagos short-eared owls. The majority of these results were comparable to the only previously reported RI for short-eared owls, calculated using a smaller sample size (*n* < 20) from animals under human care in Canada and France (Ammersbach *et al*., 2015a, 2015b). Although there was variation in the reference ranges reported from these two distinct populations, potassium was the only parameter in our study whose mean value did not fall within the previously published reference range (Galapagos short eared-owl population mean: 2.2 mmol/L, RI of short-eared owls in human care elsewhere: 3.4–6.6 mmol/L) (Ammersbach *et al*., 2015b). Values for haematology and biochemistry may vary based on geographic location and population as a complex reflection of alterations in diet, habitat and health status (Clark *et al*., 2009). The RI reported here, although blood samples were collected from owls after five to six months under managed care, still represent a large sample size from this specific unique population. We believe this resource will allow for more accurate health assessments of Galapagos short-eared owls during their time under human care and, if necessary, following their release back to Floreana.

The RI for WBC count in short-eared owls was determined based on the phyloxine-b technique, which despite being an indirect method, is considered the gold standard in avian species (Dein *et al*., 1994; Campbell and Grant, 2022b). Previous haematology reports on strigiform species utilizing this method have noted an elevated upper limit of the RI for WBC count compared to other avian species (Ammersbach *et al*., 2015a), which was consistent in this population. We also confirmed Galapagos short-eared owls to be predominately lymphocytic (mean H:L ratio of 0.6), consistent with other *Asio* species (Ammersbach *et al*., 2015a).

We were able to describe WBC morphology in this species, although basophils were degranulated in all smears. This finding is comparable to previous literature on cell morphology in Strigiformes (Ammersbach *et al*., 2015a), and is likely due to the use of an alcohol-solubilized stain known to partially dissolve or coalesce basophilic granules (Campbell and Grant, 2022a).

The Abaxis VetScan point-of-care analyser is convenient for use in this population and environment due to its small size and portability, and the low required sample volume (0.1 ml of whole blood or plasma) needed for analysis. The VetScan has clinically reliable results when compared to a reference analyser for only a subset of analytes in other avian species (Greenacre *et al*., 2008), and also specifically in Strigiformes (Ammersbach *et al*., 2015b). Therefore, while overall these results are an excellent reference baseline, future samples should consider which analyser is being used for analysis when interpreting results. The VetScan was unable to determine total protein, albumin and globulin levels in multiple samples. These errors are likely partially due to the assay performed to measure albumin, the bromcreosol green dye-binding method, which is known to be unreliable for avian samples (Greenacre *et al*., 2008; Cray *et al*., 2011; Ammersbach *et al*., 2015b). As this method was used to report the RI for albumin and globulins (as measured albumin is used to calculate the globulin concentration), use of this RI when interpreting future results should be done cautiously.

Females were noted to be significantly heavier than males in this population, consistent with previous studies of Galapagos short-eared owls (DeGroot, 1983), and some analytes were found to have statistically significant differences between male and female owls. Females had significantly higher values for packed cell volume compared to males. Alterations in PCV are occasionally noted based on sex in other avian species; however, elevated PCV has typically been noted in males and attributed to the erythropoietic effect of androgens (Clark *et al*., 2009; Chan *et al*., 2012; Pranoto and Nugrahalia, 2020). Age is more consistently noted as a significant factor when interpreting PCV results in avian species, including other owl species (Clark *et al*., 2009; Agusti Montolio *et al*., 2017). As age was not assessed during RI calculations in our study, there may have been a skewed age distribution between sexes impacting these results. Female owls were also noted to have a higher relative monocyte percentage compared to males. Monocytosis can indicate a chronic inflammatory process; however, although the relative and absolute values were statistically significantly different, the degree of difference is not suggestive of clinical significance. Female short-eared owls also had higher total protein, calcium and phosphorus levels than males. Although previous research outside the breeding season in a variety of raptor species, including short-toed snake eagles (*Circaetus gallicus*) (Baumbusch *et al*., 2021), bearded vultures (*Gypaetus barbatus*) (Hernandez and Margalida, 2010) and northern goshawks (*Accipiter gentilis*) (Hanauska-Brown *et al*., 2003), found no differences in these parameters on the basis of sex, calcium and total protein are known to fluctuate significantly in females due to hormonal changes associated with breeding and reproduction (Harr, 2002). An increase in calcium in females compared to males outside their breeding season was also reported in a study of free-living chimango caracara (*Milvago chimango*) (Paterlini *et al*., 2023). This elevated calcium level outside the time typically associated with hormonal changes and preparation for egg laying may represent alternative differences in physiology between sexes in certain species and warrants further research.

Males in this study were found to have higher concentrations of both glucose and uric acid, suggesting a difference in metabolism based on sex, though this may have been confounded by post-prandial effects and the stress of handling (Lumeij and Remple, 1991; Kimball *et al*., 2024). Increased glucose in males was also reported during the breeding season in collared scops owls (*Otus lettia*) under human care (Chan *et al*., 2012), and in two Anseriformes species outside the breeding season (Fairbrother *et al*., 1990; Pranoto and Nugrahalia, 2020).

Land birds on the Galapagos islands face a variety of threats not only from invasive species, but also from habitat loss, climate change, emerging infectious diseases including disease spillover from domestic poultry and increased human contact (Deem *et al*., 2011; Phillips *et al*., 2011; Dvorak *et al*., 2017; Jimenez *et al*., 2024; Vega-Mariño *et al*., 2024). Strategies for addressing these threats can be complicated and carry their own risks of negative impact on population health and sustainability, which only increases the need for methods to accurately assess wildlife health. Analysis of haematology and biochemistry are vital for assessing and monitoring the health of individuals and populations. The baseline reference values outlined here will be a valuable resource during continued conservation work with this unique population of short-eared owls and the species across the archipelago.

## Data Availability

The data underlying this article will be shared upon reasonable request to the corresponding author.
